# Effect of sivelestat sodium on the incidence of ventilator-associated pneumonia in patients with sepsis and ARDS

**DOI:** 10.3389/fmed.2025.1618914

**Published:** 2025-08-06

**Authors:** Xingcheng Zhang, Zhuangli Li, Xiqun Lei, Huaxue Wang, Nanbing Shan, Yun Sun

**Affiliations:** ^1^The First Department of Critical Care Medicine, The Second Affiliated Hospital of Anhui Medical University, Hefei, Anhui, China; ^2^Department of Critical Care Medicine, Fuyang Second People’s Hospital, Fuyang, Anhui, China; ^3^Department of Critical Care Medicine, The 901 Hospital of the Joint Logistic Support Force of the Chinese People’s Liberation Army, Hefei, Anhui, China; ^4^Department of Critical Care Medicine, The First Affiliated Hospital of Bengbu Medical University, Bengbu, Anhui, China

**Keywords:** sivelestat sodium, ARDS, mechanical ventilation, ventilator-associated pneumonia, sepsis, 28 days mortality

## Abstract

**Objectives:**

To evaluate the efficacy of sivelestat sodium in reducing ventilator-associated pneumonia (VAP) in patients with sepsis and acute respiratory distress syndrome (ARDS).

**Methods:**

A retrospective analysis was performed on the clinical data of 187 adult patients with sepsis combined with ARDS admitted to the intensive care unit (ICU) of Fuyang Second People’s Hospital from 1 January 2022 to 1 December 2024. Among these patients, 60 received sivelestat sodium as part of their treatment, while 127 did not. The treatment efficacy indices were oxygenation index (PaO_2_/FiO_2_), procalcitonin (PCT), C-reactive protein (CRP), and interleukin-6 (IL-6) levels (all measured before and within 7 days of treatment), and VAP, bacteremia, time to first mechanical ventilation, CRRT, length of stay in ICU, length of stay, and 28 days mortality.

**Results:**

There were no significant differences in age, sex, comorbidities, use of hormones, use of vasoactive drugs, APACHE II score, or SOFA score between the two groups before treatment. Compared with patients who did not receive sivelestat sodium, those treated with sivelestat sodium had significantly lower incidences of VAP (χ^2^ = 6.910, *P* = 0.009) and bacteremia (χ^2^ = 5.372, *P* = 0.023), as well as shorter times to first mechanical ventilation (t = −2.071, *P* = 0.041) and ICU stays (t = −2.085, *P* = 0.039). At 28 days, the fatality rate in the sivelestat group was 33.33%, and that in the control group was 34.65%, although this slight reduction was not significant (χ^2^ = 0.031, *P* = 1.000). There was also no significant difference in the length of stay between the two groups (t = −0.609, *P* = 0.054). Log-Rank test analysis revealed that the time without VAP in the sivelestat group was significantly longer than that in the control group (χ^2^ = 7.600, *P* = 0.006). After adjusting for APACHE II score and age, COX proportional risk model analysis revealed that the 28 days survival risk for VAP with sivelestat sodium was 34.67% higher than that in the control group (Z = −2.537, *P* = 0.011).

**Conclusion:**

Sivelestat sodium therapy was associated with a reduced incidence of VAP and a shorter ICU stay in patients with ARDS. However, there was no significant benefit on 28 days survival or total hospital stay.

## 1 Introduction

Sepsis is a life-threatening organ dysfunction caused by the host’s disproportionate response to infection (1). In severe cases, sepsis can rapidly progress to septic shock, a complication that affects multi-system organs simultaneously, and which because of its high incidence, high mortality, and high economic cost, is a major global public health concern (2, 3). In patients with severe sepsis, the incidence of acute respiratory distress syndrome (ARDS) is 25%–50%, although its incidence increases with further aggravation of sepsis (4, 5). Because of its complexity, the pathogenesis of sepsis remains unclear. One possible mechanism of organ damage in sepsis is related to neutrophil elastase (NE). NE has already been demonstrated to be an important pathogenic factor in sepsis complicated with ARDS, acute kidney injury, or myocardial injury (4–6). With these considerations in mind, inhibition of NE has been attempted in animal experiments to reduce organ damage associated with sepsis (7, 8).

Sivelestat sodium (an NE inhibitor) was reported to be effective and safe in the treatment of sepsis with ARDS (9, 10). However, its role in the prevention of ventilator-associated pneumonia (VAP) has not been reported. To improve patient outcome in sepsis combined with patients with ARDS, we investigated the effect of sivelestat sodium on sepsis combined with ARDS patients in ICU, and its influence on the incidence of inflammatory cells and VAP in patients. These results should provide a reference for future treatment of sepsis patients in ICU.

## 2 Data and methods

### 2.1 Baseline data

This retrospective study included 187 patients with sepsis combined with ARDS who were admitted to the ICU of Fuyang Second People’s Hospital from January 2022 to December 2024. All subsequent treatment decisions were under the instruction of the attendant medical staff (with informed consent). Demographic and clinical data were collected, including age, sex, comorbidities, use of hormones, use of vasoactive drugs, continuous renal replacement therapy (CRRT), APACHE II score within 24 h of ICU admission, and SOFA score. Patients were classified retrospectively into two groups according to whether they had received sivelestat sodium treatment or not.

### 2.2 Inclusion and exclusion criteria

Inclusion criteria: (1) A diagnosis of sepsis or septic shock that met sepsis 3.0 criteria [infection or suspected infection + sequential organ failure assessment (SOFA) score ≥ 2 (1)] and was in line with the new global diagnostic criteria for ARDS 2023 (11); (2) Aged ≥ 18 years old (of either gender). (3) A hospitalization period ≥ 7 days; (4) Mechanical ventilation treatment was performed (ventilation time > 48 h).

Exclusion criteria: (1) An obvious absence of case data; (2) A malignant tumor diagnosis; (3) Previous chronic severe lung disease or heart disease.

### 2.3 Ethics

The research design adopted was that of a retrospective study. Laboratory indicators of patients were only collected anonymously, and no intervention with the research drug was conducted. This study was reviewed for approval by the ethics committee overseeing our hospital’s scientific research projects and was deemed to meet all medical ethics requirements (Ethics review number: 20240523024).

### 2.4 Methods

Patients were retrospectively classified into a sivelestat treatment group and a control group based on whether they received sivelestat within 7 days. Other supportive treatments were in-line with the Save Sepsis Campaign: International Guidelines for the Management of Sepsis and Septic Shock 2021 (12, 13). Common supportive treatments included: combination antimicrobial therapy, fluid therapy, vasoactive and positive inotropic drugs, analgesic sedation, and (as and when required) standard treatment for acute respiratory distress syndrome (ARDS), including low tidal volume lung protection ventilation, lung re-expansion, and prone ventilation.

The protocol used to administer sivelestat sodium (Suzhou Erye Pharmaceutical Co., LTD. National Drug Approval No. H20203093 0.1 g) was as follows. In line with recommendations, sivelestat sodium was administered within 24–72 h after the onset of the disease. A 24 h dose of sivelestat sodium (calculated from the patient’s body weight using 4.8 mg/kg) was diluted with 250–500 mL of normal saline. The drug solution was initially drawn with a 50 mL syringe and diluted to a total volume of 48 mL. The drug delivery flow rate was set at 2 mL/h using an intravenous drip micropump, and the drug was then administered intravenously over 24 h (equivalent to 0.2 mg⋅kg^–1^⋅h^–1^). As an alternative, the daily dose was formulated in three doses for continuous administration by intravenous drip for 8 h. The longest course of administration was 14.0 days (14).

### 2.5 Clinical observation indicators

The following data were obtained from the hospital healthcare system within 24 h of admission to the ICU: age; sex; comorbidities including Hypertension, Diabetes, Coronary heart disease (CHD), Liver insufficiency, kidney insufficiency, etc.; acute physiological and Chronic Health Assessment II (APACHE II) scores; hormone (Hydrocortisone) use and vasoactive drug (Norepinephrine, adrenaline) use; arterial blood gas analysis results; laboratory indicators; mechanical ventilation; and CRRT use. For all included patients, CRP, PCT, IL-6, and oxygenation index values were extracted from the medical system on days 1 (D1), 3 (D3), and 7 (D7).

The two groups were subsequently compared for VAP, bacteremia, 28 days fatality rate (including terminal abandonment), first mechanical ventilation time, ICU stay time, etc. The VAP diagnosis was based on the 2018 Chinese Adult Hospital acquired Pneumonia and VAP diagnosis and treatment guidelines (15). All patients presenting positive blood culture were judged to be combined with bacteremia.

### 2.6 Endpoints

The primary endpoint of this retrospective study was the incidence of VAP. Secondary endpoints included time to first mechanical ventilation, 28 days mortality, length of hospital stay, length of ICU stay, oxygenation index value, and inflammatory markers (including CRP, PCT, and IL-6).

### 2.7 Statistical methods

Data were analyzed using SPSS 25.0 and R 4.4.1. Continuous variables with normally distributed are presented as mean ± standard deviation (SD) and were compared using independent samples *t*-tests. Continuous variables with non-normally distributed are presented as median (with interquartile range) [M(Q25, Q75)] and were compared using Mann-Whitney U tests. Categorical data were analyzed using Chi-square tests. To assess whether sivelestat sodium reduced the incidence of VAP, survival analysis was performed using Log-rank tests and Cox proportional hazards regression models. To assess statistical significance, a *P*-value < 0.05 was used.

## 3 Results

### 3.1 Comparison of baseline data

After careful consideration of the inclusion and exclusion criteria, a total of 187 patients were included in this study: sivelestat sodium group, 60 patients; Control group, 127 patients. There was no statistically significant difference in gender, age, or comorbidities between the two groups (*P* > 0.05). Furthermore, a comparison of APACHE II scores, SOFA scores, and treatment at admission revealed that there was no statistically significant difference between these variables across the two groups (*P* > 0.05). As demonstrated in Table 1, the two groups can be considered comparable for all confounding factors.

**TABLE 1:** Comparison of baseline data between the two groups.

Variable	Sivelestat sodium group (*n* = 60)	Control group (*n* = 127)	Test value	*P-*value
Age (mean ± SD)	62.2 ± 17.023	63.1 ± 17.023	−0.341	0.734
≤ 75 (age, %)	48 (80)	100 (78.74)	0.039	1.000
Sex (male, %)	47 (78.33)	93 (73.23)	0.564	0.477
**Complication (%)**				
Hypertension	10 (16.67)	22 (17.32)	0.012	1.000
Diabetes	12 (20)	16 (12.59)	1.753	0.194
Coronary heart disease (CHD)	13 (21.67)	19 (14.96)	1.292	0.299
Liver dysfunction	28 (46.67)	54 (42.52)	0.285	0.637
Renal insufficiency	26 (43.33)	43 (33.86)	1.571	0.256
Disorder of blood coagulation function	15 (25)	29 (22.83)	0.106	0.854
Gastrointestinal bleeding	6 (10)	15 (11.81)	0.134	0.808
CRRT (%)	21 (35.00)	49 (38.58)	0.223	0.746
**Hormone use (%)**				
Hydrocortisone (200 mg/d)	23 (38.33)	43 (33.86)	0.357	0.623
**Vasoactive drug use (%)**				
Norepinephrine	51 (85.00)	116 (91.34)	1.714	0.210
Adrenaline	10 (16.67)	28 (22.05)	0.729	0.442
APACHE II score (mean ± SD)	21.85 ± 6.571	21.42 ± 9.971	0.353	0.724
SOFA score (mean ± SD)	10.50 ± 5.87	9.17 ± 5.09	1.582	0.115

### 3.2 Changes in inflammatory indicators and oxygenation index

**FIGURE 1:**
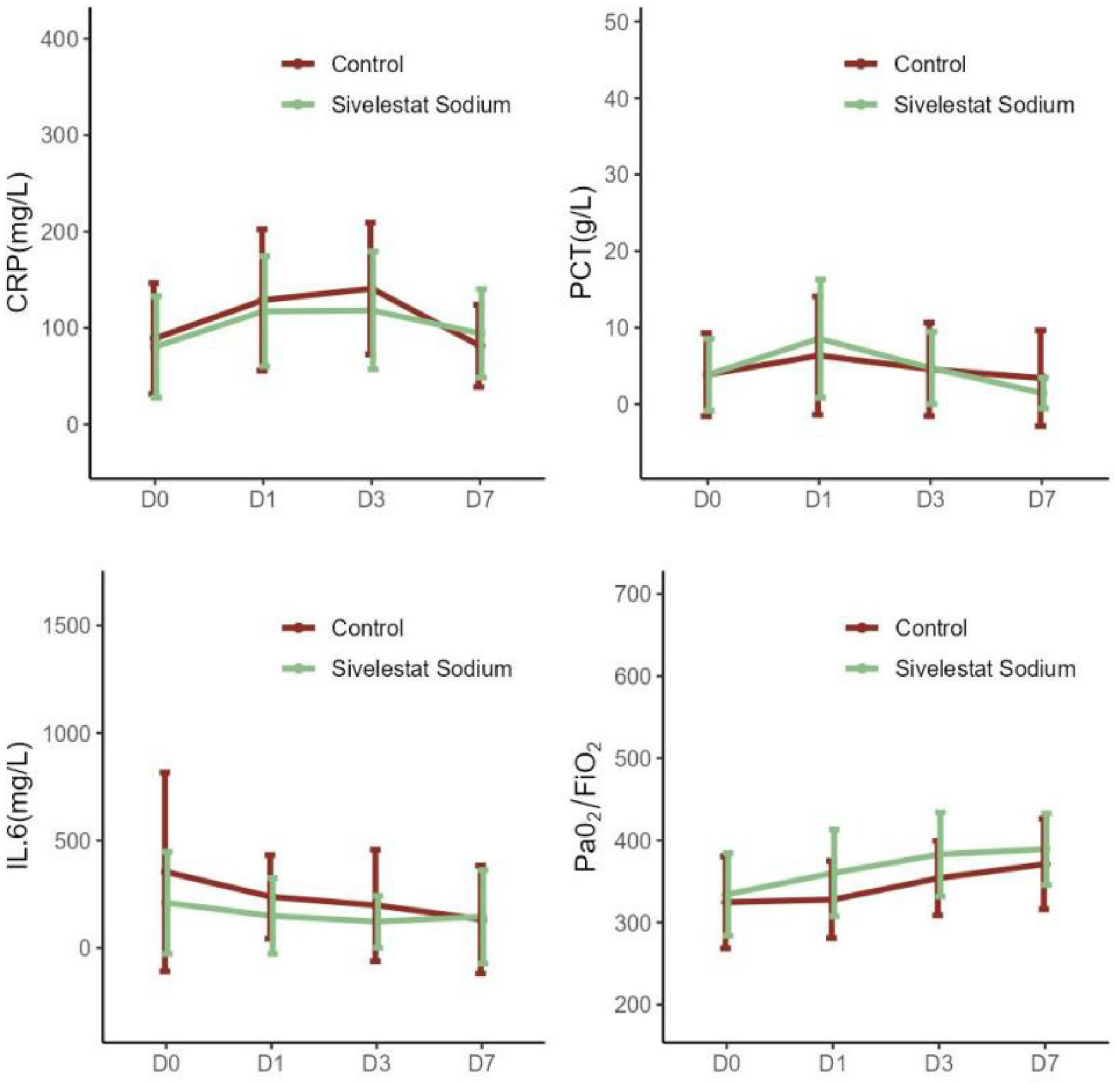
Changes in infection index and oxygenation index in sepsis patients with acute respiratory distress syndrome (ARDS).

A comparison of CRP, PCT, and IL-6 levels in the sivelestat sodium group and the control group from baseline to D1, D3, and D7 revealed statistically significant differences between the groups (at D7, *P* = 0.042, *P* = 0.041, and *P* = 0.009, respectively). By day 7, the oxygenation index of the sivelestat sodium group had increased (compared with baseline) to 55.25 (95% CI, 37.99–72.50631), while that of the control group had only increased (compared with baseline) to 46.481 (95% CI, 33.40–59.56), and this difference was statistically significant (*P* < 0.05; Table 2; Figure 1).

**TABLE 2:** Effect of sivelestat sodium on oxygenation index and inflammation indicators.

Variable	Change from baseline (95% CI)*		*P*
	Sivelestat group (*n* = 60)	Control group (*n* = 127)	
CRP (mean ± SD)*			
D1	37.02 ± 49.562	−23.1676 ± 86.26	0.733
D3	37.92 ± 65.847	51.72 ± 78.503	0.210
D7	14.09 ± 65.218	−7.71 ± 73.446	0.042
PCT (mean ± SD)*			
D1	4.72 ± 7.482	2.49 ± 8.107	0.065
D3	0.9 ± 5.058	0.69 ± 7.3	0.818
D7	−2.39 ± 5.002	−0.48 ± 7.575	0.041
IL-6 (mean ± SD)*			
D1	−61.06 ± 263.377	−116 ± 431.6	0.283
D3	−87.46 ± 248.586	−155.58 ± 431.091	0.172
D7	−65 ± 296.448	−221.62 ± 512.897	0.009
**PaO_2_/FiO_2_ (mean ± SD)**			
D1	26.08 ± 72.336	3.29 ± 71.829	0.046
D3	48.78 ± 73.03	29.48 ± 71.403	0.091
D7	55.25 ± 66.8	46.48 ± 75.071	0.421

*CI, confidence interval; CRP, C-reactive protein; PCT, procalcitonin; IL-6, Interleukin-6.

### 3.3 Comparison of clinical indicators

The incidence of VAP in the control group was 29.13% (37 cases), while the incidence of VAP in the sivelestat sodium group was significantly lower (7 cases). 11.67%. The incidences of VAP and bacteremia in the sivelestat sodium group were reduced (compared with the control group), and these differences were both statistically significant (χ^2^ = 6.910, *P* = 0.009 and χ^2^ = 5.372, *P* = 0.023, respectively). Conversely, there were no significant differences in 28 days mortality or length of stay between the two groups (χ^2^ = 0.031, *P* = 1.000 and t = −0.609, *P* = 0.054, respectively). However, the first mechanical ventilation time and ICU stay time of the sivelestat sodium group were shorter than those of the control group (t = −2.071, *P* = 0.041 and t = 0.139, *P* = 0.039, respectively; see Table 3).

**TABLE 3:** Comparison of clinical indicators.

Variable	Sivelestat sodium group (*n* = 60)	Control group (*n* = 127)	Test value	*P*
VAP (%)	7 (11.67)	37 (29.13)	6.910	0.009
Bacteremia (%)	15 (25.00)	54 (42.52)	5.372	0.023
First mechanical ventilation time (mean ± SD)	7.00 (4.00, 12.50)	6.00 (9.00, 14.00)	−1.999	0.046
ICU stay (mean ± SD)	10.00 (6.00, 18.00)	11.50 (7.00, 20.00)	−2.105	0.035
Length of stay (mean ± SD)	16.50 (9.25, 27.00)	17.00 (9.00, 29.00)	−0.048	0.962
28 days mortality (%)	20 (33.33)	44 (34.65)	0.031	1.000

### 3.4 VAP occurrence analysis

**FIGURE 2:**
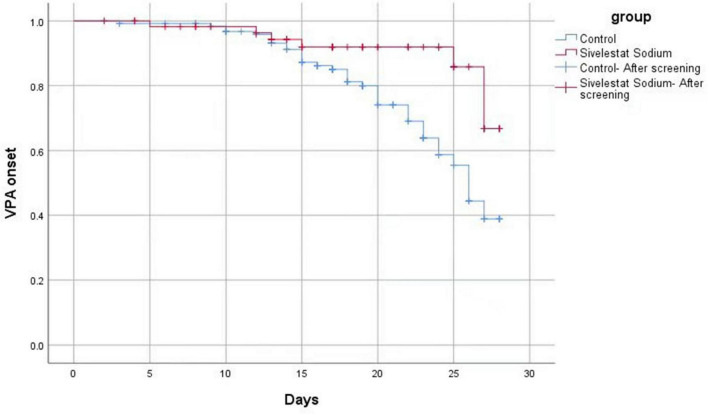
Log-Rank test analysis results.

A Log-Rank test analysis revealed that VAP free time in the sivelestat group was longer than VAP free time in the control group (Figure 2), and this difference was statistically significant (χ^2^ = 7.600, *P* = 0.006). After adjusting for APACHE II score and age, a COX proportional risk model analysis established that the survival risk for patients in the sivelestat sodium group was significantly lower than that for the control group (hazard ratio, 0.282). Hence, the survival risk in patients treated with sivelestat sodium was 34.67% higher than in the control group (Z = −5.2.537, *P* = 0.011).

### 3.5 Comparison of 28 days survival rate between the two groups

**FIGURE 3:**
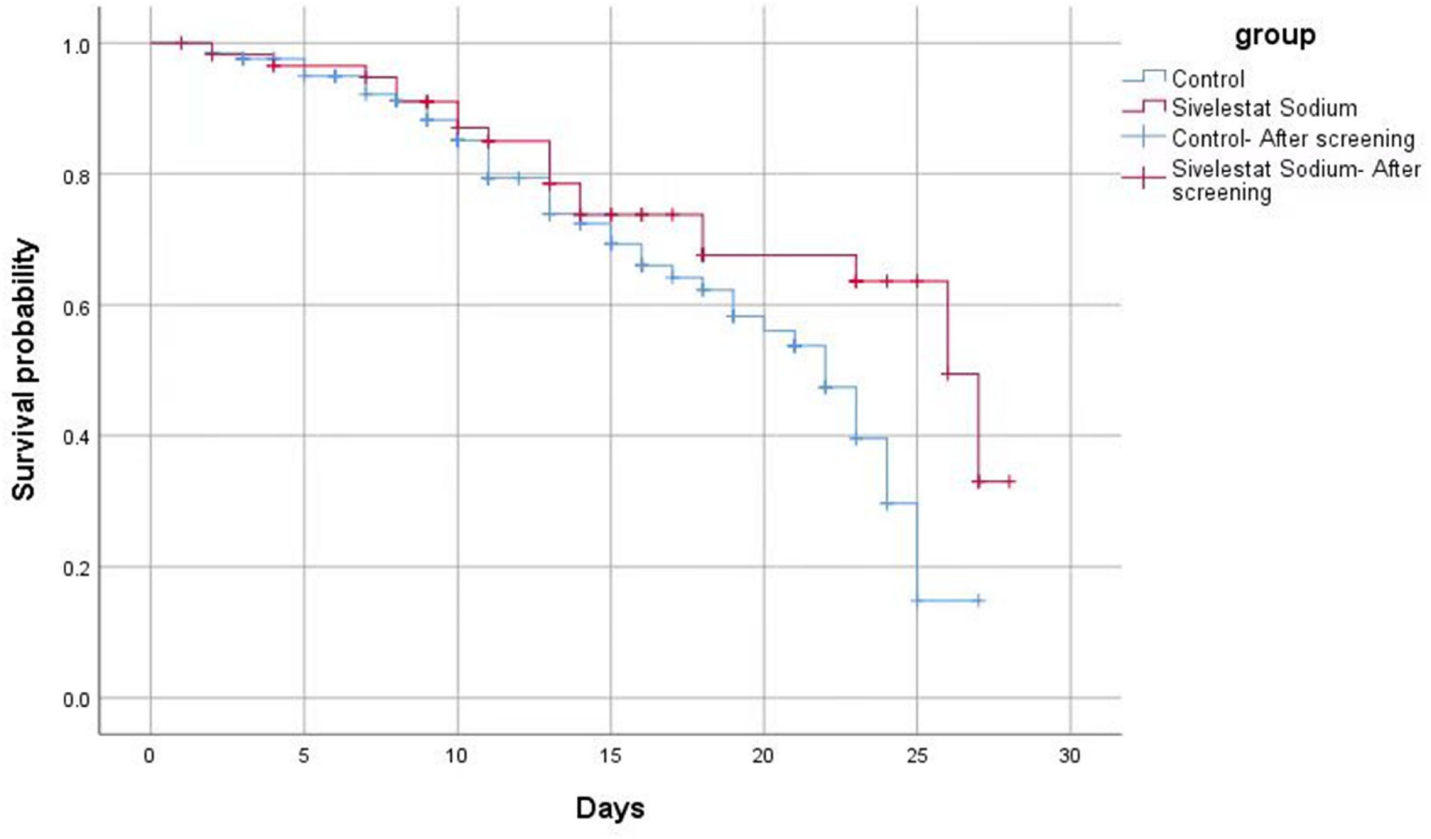
The mortality of patients in the sivelestat sodium group exhibited a decreasing trend (compared with the control group). However, the difference between the two groups was not statistically significant.

Of the 187 cases included, 62 patients died, an overall case fatality rate of 33.16%. Of the 60 cases in the sivelestat sodium group, 20 patients died, yielding a case fatality rate of 33.33%. Of the 127 cases in the control group, 44 patients died, yielding a fatality rate of 34.65%. While the 28 days fatality rate of the sivelestat sodium group did exhibit a decreasing trend (Figure 3), the difference between the two groups was not statistically significant (χ^2^ = 4.100, *P* = 0.040).

## 4 Discussion

Sepsis patients with ARDS often require endotracheal intubation and invasive mechanical ventilation. However, prolonged mechanical ventilation is associated with an increased risk of VAP (16). When ARDS is complicated by VAP, the accumulation of inflammatory secretions in the airways may obstruct airflow, thereby exacerbating ARDS and impairing the patient’s oxygenation function (17, 18). Furthermore, VAP has been demonstrated to prolong the duration of mechanical ventilation (MV) and ICU stay, increase healthcare costs, significantly worsen patient prognosis, and increase mortality risk (17, 18). Interestingly, recent studies have indicated that inhibition of NE may improve clinical outcomes in patients with acute lung injury (ALI) or ARDS (19), and also enhance weaning success rates and ICU discharge rates (20). Therefore, in the treatment of sepsis complicated with ARDS, application of the NE inhibitor sivelestat sodium via injection may reduce the incidence of VAP (and related risks) by shortening the duration of mechanical ventilation and ICU stay.

According to research data obtained from domestic and international studies, the overall incidence of VAP in patients undergoing mechanical ventilation ranges from 5% to 40%. In populations with sepsis complicated by ARDS, the incidence of VAP can be as high as 29% (20, 21). Considering the clinical prognosis for patients with sepsis complicated by ARDS, VAP is associated with an all-cause mortality rate of 30%–70%, with attributable mortality (i.e., deaths directly caused by VAP) accounting for 20%–30%. When the infecting pathogens are multidrug-resistant bacteria (MDR) or pan-drug-resistant bacteria (PDR), patient mortality rates increase further to between 38.9% and 76% (20, 21). In the present study, we found that the incidence of VAP in the control group was 29.13% (37 cases), which is consistent with previous research findings. Conversely, the incidence of VAP in the sivelestat sodium group was significantly decreased (seven cases; 11.67%). This notable difference suggests that sivelestat sodium may effectively reduce the risk of developing VAP in patients with sepsis complicated by ARDS through its unique pharmacological mechanisms, demonstrating promising clinical application prospects.

Additionally, treatment with sivelestat sodium significantly reduced the duration of both initial mechanical ventilation and ICU stay in patients with sepsis complicated by ARDS (compared to the control group). Several earlier studies provide additional support for this finding. A meta-analysis including 16 RCTs, cohort studies, and large-sample studies (totaling 9,202 patients) confirmed that sivelestat sodium helps improve the oxygenation index and shortens the duration of mechanical ventilation in patients with mild to moderate ARDS (22). An observational study from Japan on acute lung injury following aspiration also indicated that patients treated with sivelestat sodium had a lower cumulative proportion requiring mechanical ventilation within the first 14 days (23). These findings provide support for the results presented here, confirming that sivelestat sodium helps reduce patients’ dependence on mechanical ventilation and shortens the disease course (compared to conventional treatment). Reducing the duration of mechanical ventilation and hospitalization holds significant clinical implications. Previous research has shown that the incidence of VAP increased markedly with prolonged mechanical ventilation, rising from 5% on the first day to 65% by the 30^th^ day (24). Prolonged mechanical ventilation has been shown to damage the respiratory mucosa, increasing the risk of pathogen invasion, while prolonged ICU stays increase the risk of environmental exposure and medical interventions, thereby raising the likelihood of cross-infection. An ICU stay exceeding 7 days or mechanical ventilation lasting more than 7 days typically indicates critical illness, with compromised immune and respiratory defense functions, predisposing patients to recurrent infections and increasing the risk of infections with dangerous nosocomial pathogens such as carbapenem-resistant *Acinetobacter baumannii* (CRAB) (25). Therefore, by reducing dependence on mechanical ventilation and shortening ICU stays, sivelestat sodium not only accelerates patient recovery but may also help lower the incidence of secondary infections like VAP.

Moreover, our results also provide evidence that sivelestat sodium can significantly improve inflammatory indicators (including IL-6, PCT, CRP) and oxygenation function (PaO_2_/FiO_2_ ratio) in patients with sepsis complicated with ARDS. The underlying mechanism of action of sivelestat sodium in these processes is likely related to its effective inhibition of excessive inflammatory responses and alleviation of lung injury (26, 27). From a pathophysiological perspective, IL-6 is a key pro-inflammatory factor that stimulates the release of other inflammatory mediators (e.g., TNF-α and IL-1β) and increases pulmonary vascular permeability, eventually leading to alveolar and interstitial edema, which ultimately deteriorates oxygenation function further. CRP and PCT are important prognostic markers for sepsis and ARDS, and changes in their levels can reflect the severity of the disease and the therapeutic effect. Multiple clinical studies have demonstrated the anti-inflammatory and oxygen-improving effects of sivelestat sodium. A randomized double-blind controlled trial demonstrated that sivelestat sodium could significantly reduce the levels of various inflammatory mediators (including IL-6) in patients (28). Similar anti-inflammatory effects have also been documented in studies on COVID-19 patients in China. Notably, early application of sivelestat sodium in patients with sepsis complicated with ARDS (within 48 h after diagnosis) significantly improved their organ dysfunction score and oxygenation index, and reduced the associated mortality rate (29, 30). In patients with acute lung injury, sivelestat sodium more effectively reduced IL-6, TNF-α, and CRP levels (in comparison with doxofylline), and significantly increased the oxygenation index at 24 and 72 h (31). Furthermore, case reports indicated that three patients with chemical inhalation-induced lung injury presenting with SpO_2_ < 92% had significantly improved oxygenation function and a good prognosis after receiving sivelestat sodium combined with nasal high-flow oxygen therapy (10). Although further research is still needed on the efficacy differences of NE inhibitors in different ARDS subtypes at present, the existing evidence indicates that NE inhibitors (such as sivelestat sodium) can exert potential pulmonary protective effects by down-regulating the levels of inflammatory markers such as IL-6 and CRP (19). These findings provide an important theoretical basis and clinical reference for the application of sivelestat sodium in the treatment of sepsis complicated with ARDS.

A Log-Rank test and COX proportional hazards model analysis were used to confirm that sivelestat sodium could significantly reduce the incidence of VAP in patients with sepsis complicated with ARDS. The underlying mechanism of action is postulated to involve multiple pathophysiological processes. Firstly, during the progression of sepsis-related ARDS, the degradation of extracellular matrix proteins mediated by NE can lead to alveolar injury and promote the formation of emphysema and subepithelial fibrosis (32). Sivelestat sodium alleviates this neutrophil-mediated endothelial injury by reducing plasma NE concentration, ultimately protecting the integrity of alveolar epithelial cells and pulmonary micro-vessels and improving oxygenation function (14, 33); Secondly, sivelestat sodium can regulate the complex immune response and cellular signal transduction process in sepsis-related ARDS. Indeed, early application of sivelestat sodium effectively inhibits the progression of inflammatory response and tissue damage. Additionally, sivelestat sodium is reported to improve gastrointestinal dysfunction in patients with sepsis and alleviate inflammatory dysbiosis (34). Shimizu et al. demonstrated that sivelestat sodium reduces the incidence of VAP by enhancing the tight junctions of endothelial cells, inhibiting the production of endotoxins in the intestine and the migration of microbiota (27). Through a combination of these mechanisms, sivelestat sodium treatment can effectively improve the lung function of patients with sepsis complicated with ARDS and significantly shorten the duration of mechanical ventilation and the length of stay in the ICU (25).

In patients with sepsis complicated with ARDS, sivelestat sodium treatment significantly improved the inflammatory indicators (IL-6, PCT, CRP) and the oxygenation index (PaO_2_/FiO_2_), and significantly reduced the duration of mechanical ventilation time and ICU stay, and the incidence of VAP. However, we did not observe any significant improvement in the overall prognosis for this cohort of patients (e.g., in the 28 days mortality rate). Similar results have been reported in several recent studies (9, 29). For instance, a number of small-sample clinical studies conducted in China and abroad from 2006 to 2022 consistently demonstrated that, even though it could increase the oxygenation level of patients with ARDS, reduce the duration of mechanical ventilation, reduce the length of stay in the ICU, and reduce medical expenses, sivelestat sodium failed to significantly improve the survival rate of patients (32, 35). However, a large-sample retrospective study based on the national database of Japan in 2017 (including 1997 patients with ARDS treated with sivelestat sodium and 2,279 patients who did not receive treatment) showed that the use of this drug within 7 days of admission could significantly reduce the mortality rate (within a 3 months period following treatment) of these patients (36). A meta-analysis published in 2023 reported that sivelestat sodium could improve the PaO_2_/FiO_2_ levels of ARDS patients, shorten the duration of mechanical ventilation and ICU stay, and also reduce the mortality rate (37). Possible explanations for these differences are: Firstly, the patients included in this study were in a more critical condition (all combined with sepsis and ARDS), and some patients failed to receive treatment within the optimal treatment time window (24–72 h after onset) (34); Secondly, the small sample size limits the statistical power. Although the mortality rate of the sivelestat sodium group was lower than that of the control group, the difference was not statistically significant. Additional studies are required to further explore the impact of medication timing (especially early application) on clinical prognosis.

The pathogenesis of ARDS complicated with VAP involves the interaction of multiple factors, including the pathological process of ARDS and the impaired immune function caused by the primary disease, the risk of cross-infection in the ICU environment, and secondary pulmonary parenchymal infection under the background of broad-spectrum antibiotic use. Together, these factors contribute to a significant increase in the difficulty of treatment and an increase in the risk of patient death. Due to high heterogeneity in the etiological characteristics of the disease, in its severity, and in the clinical progression of patients, a unified clinical treatment standard has not yet been formulated. Considering the limitations of the existing evidence, more reliable treatment norms should be established in the future, preferably through large-scale, multicenter, randomized controlled trials combined with long-term follow-up studies (recommended ≥ 90 days) (38).

Shortcomings of this study: (1) As a single-center study with a small sample size, some potential confounders may not be evaluated; (2) No limit was set on the use of other anti-inflammatory drugs in addition to sivelestat sodium. Some patients were co-administered multiple anti-inflammatory drugs, which may affect the efficacy of sivelestat sodium or confound the results. Additional studies are needed to test the effects of other anti-inflammatory drugs and sivelestat sodium on patients with ARDS complicated by sepsis.

## 5 Conclusion

In conclusion, the results presented here provide firm evidence that sivelestat sodium treatment is associated with a reduced incidence of VAP in patients with sepsis complicated with ARDS under mechanical ventilation. Nonetheless, despite these positive results, we did not observe a significant benefit in the 28 days survival rate or the total length of hospital stay.

## Data Availability

The raw data supporting the conclusions of this article will be made available by the authors, without undue reservation.
